# Supplementary figures and images for: Targeting Nup358/RanBP2 by a viral protein disrupts stress granule formation
Source: PLoS Pathog. 2022 Dec 1;18(12):e1010598. doi: 10.1371/journal.ppat.1010598 (PMC9746944; doi:10.1371/journal.ppat.1010598)

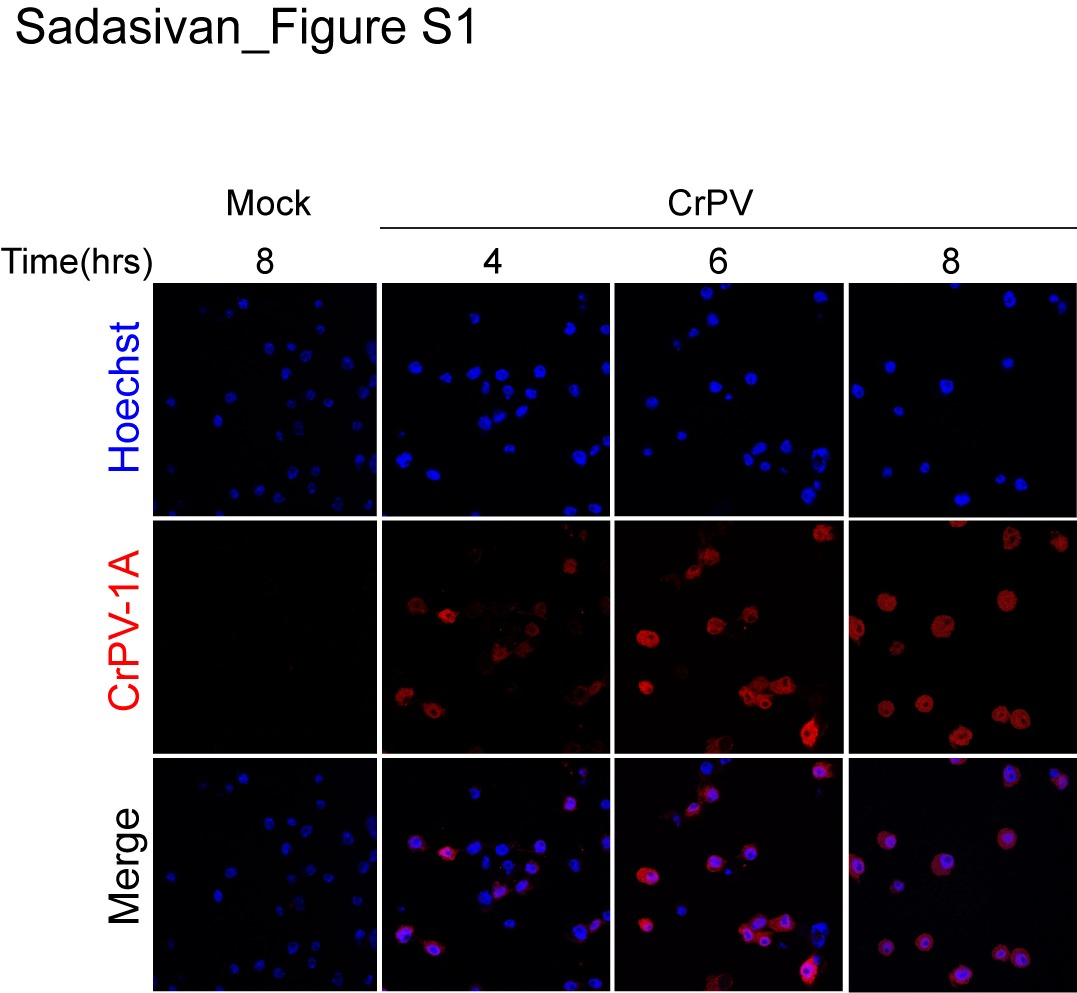

Supplement: S1 Fig — (A) Fluorescent images of S2 cells infected with CrPV (MOI 10) at indicated time points stained with CrPV-1A antibody (red) or Hoechst dye (blue). (TIF) [file ppat.1010598.s001.tif]

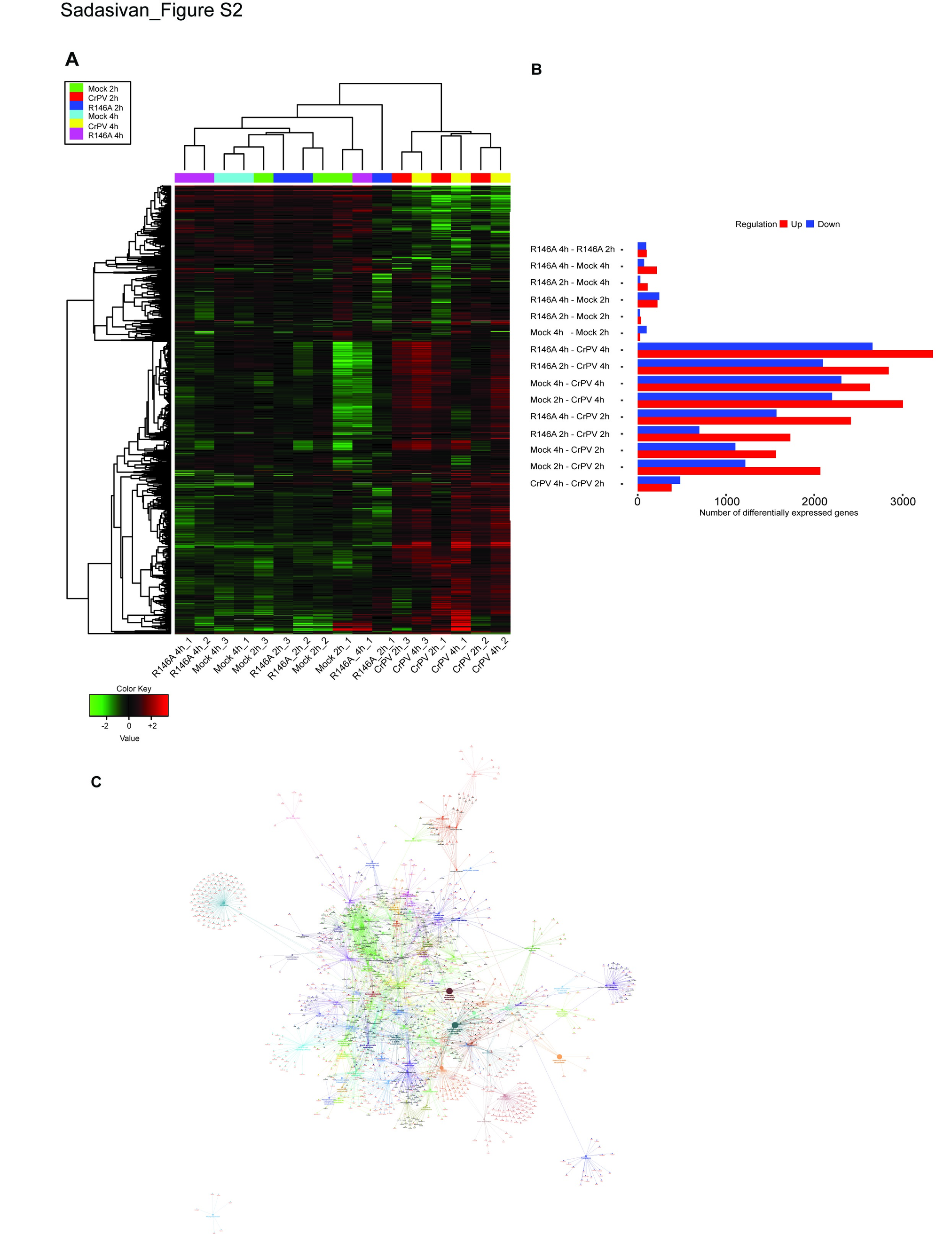

Supplement: S2 Fig — CrPV infection induces changes in gene expression (A) Hierarchical clustering of top 1000 genes (ranked by standard deviation across all the samples) showing difference in gene expression induced during virus infection. (B) Bar diagram showing comparisons on number of differentially expressed genes (C) Network analysis on upregulated genes. (TIF) [file ppat.1010598.s002.tif]

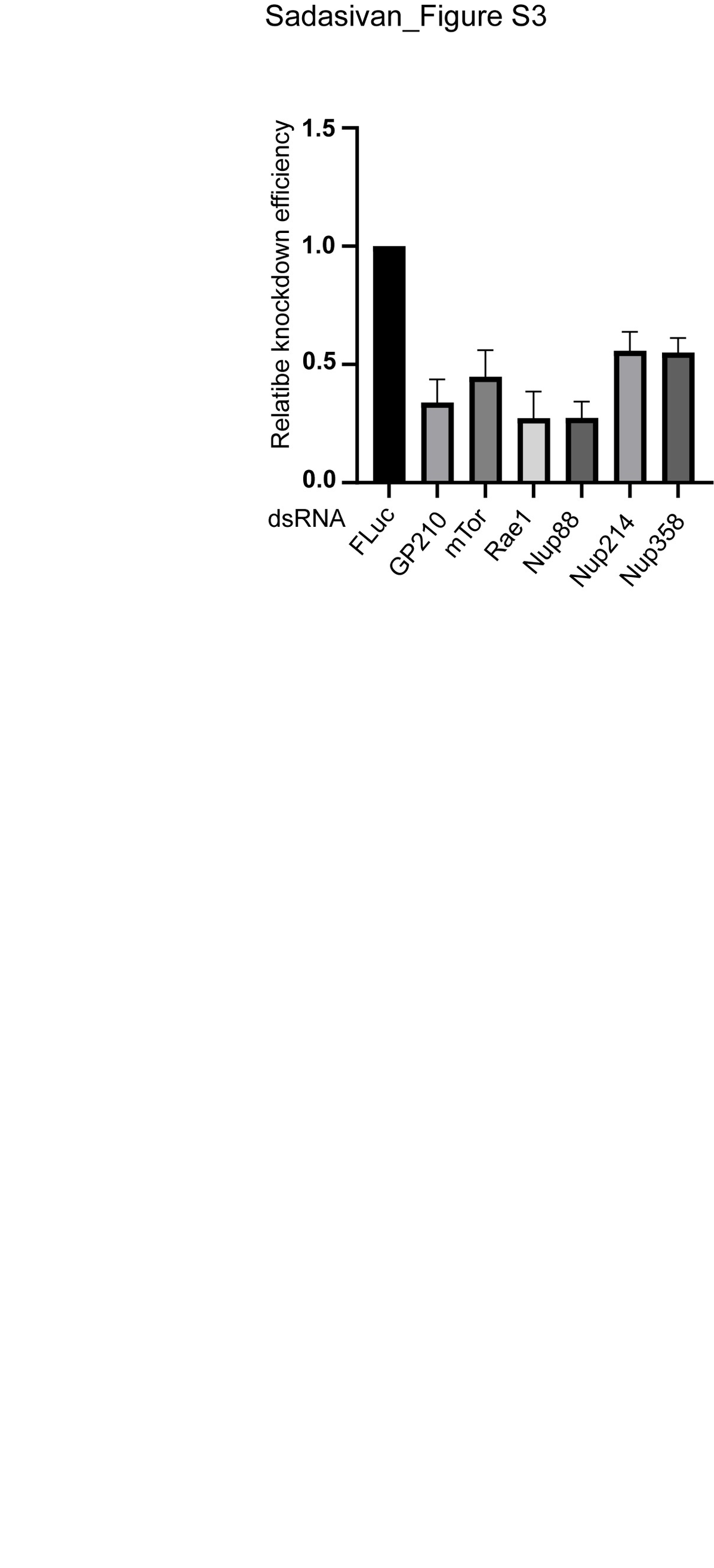

Supplement: S3 Fig — Bar graph showing relative mRNA levels by qRT-PCR in S2 cells treated with indicated dsRNAs normalized to Rps9 mRNA levels. Data are mean ± SD from three independent experiments. (TIF) [file ppat.1010598.s003.tif]

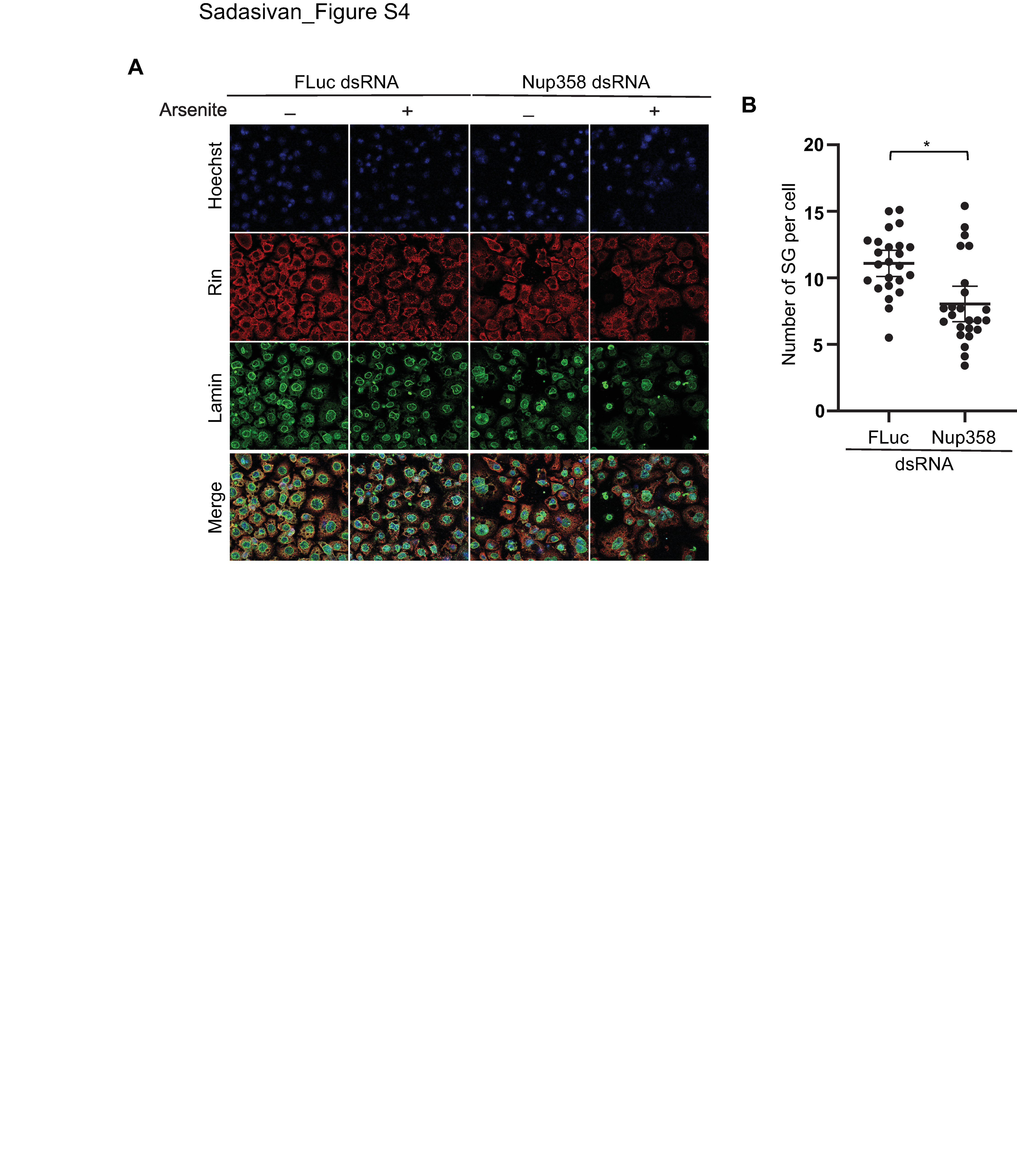

Supplement: S4 Fig — Antibody staining of Rin (red) or Lamin (green) of S2 cells treated with control dsRNA or Nup358 dsRNA in the presence or absence of arsenite. Hoechst staining is shown in blue. (B) Box plot of the number of Rin foci per cell. At least 30 cells were counted for each condition from two independent experiments. Data are mean ± SD. p < 0.021(*) by a one-way ANOVA (nonparametric) with a Bonferroni’s post hoc-test. (TIF) [file ppat.1010598.s004.tif]

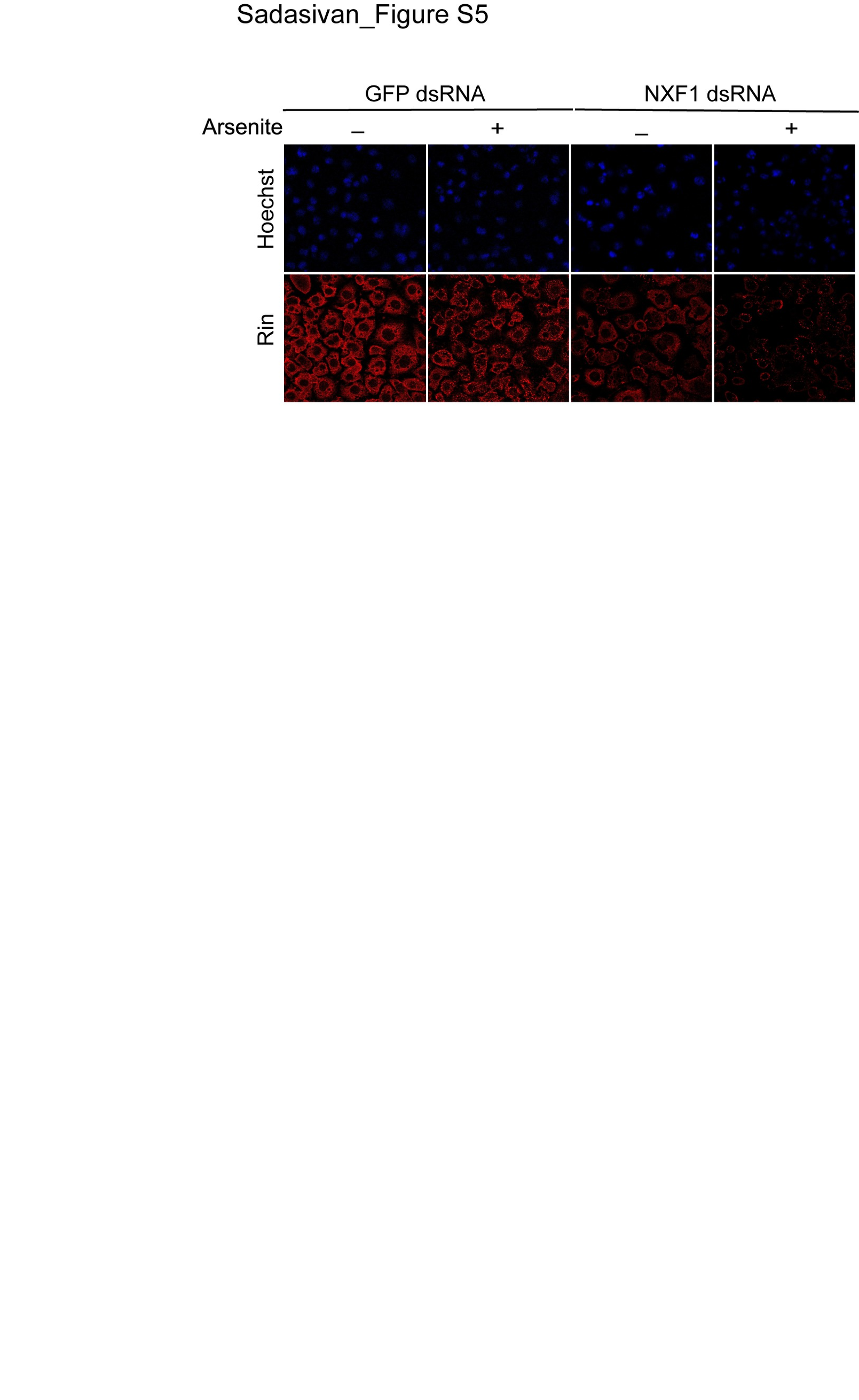

Supplement: S5 Fig — Antibody staining of Rin (red) of S2 cells treated with control dsRNA or NXF1 dsRNA (for 72 hrs) followed by one-hour treatment in the presence or absence of 500 μM sodium arsenite. Hoechst staining is shown in blue. (TIF) [file ppat.1010598.s005.tif]

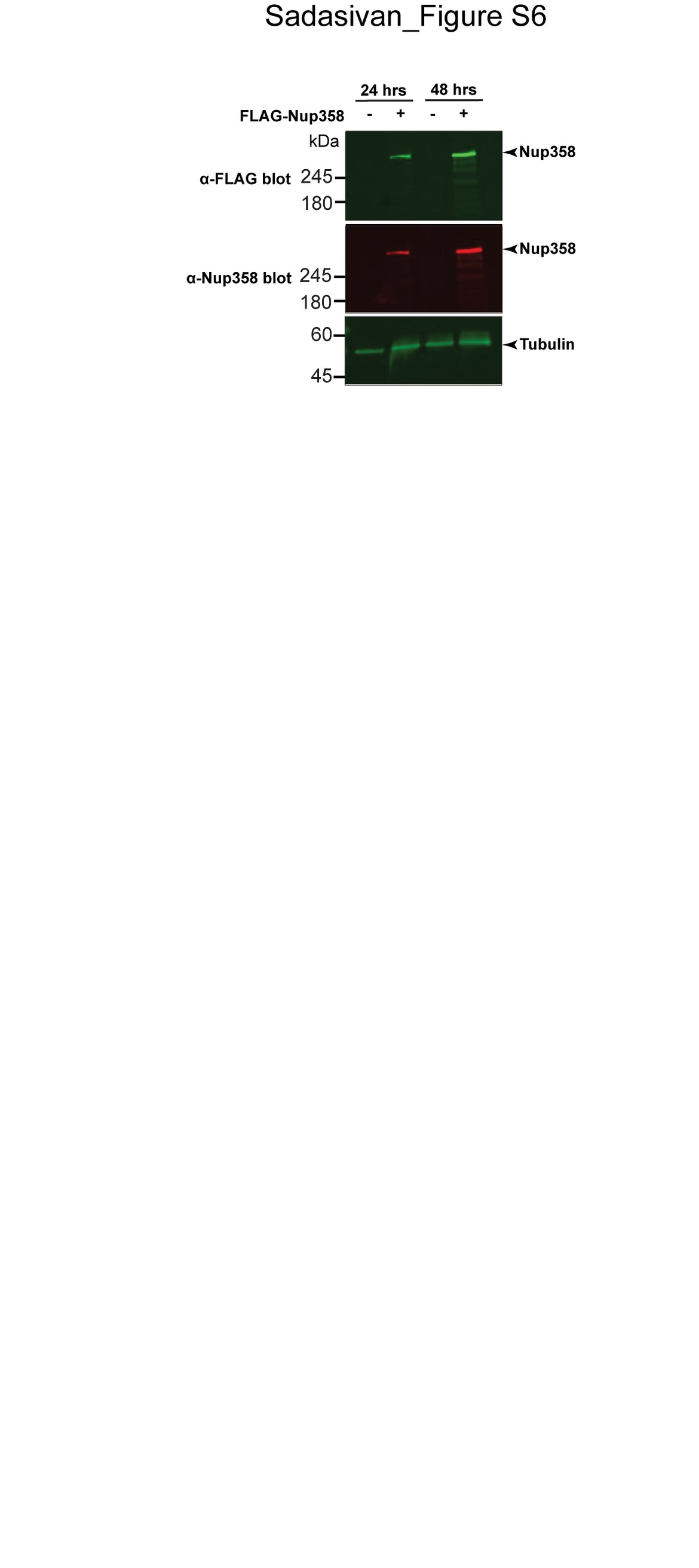

Supplement: S6 Fig — Immunoblot of S2 cell lysates transfected with. (TIF) [file ppat.1010598.s006.tif]
