# Supplementary material for: Targeting Nup358/RanBP2 by a viral protein disrupts stress granule formation
Source: PLoS Pathog. 2022 Dec 1;18(12):e1010598. doi: 10.1371/journal.ppat.1010598 (PMC9746944; doi:10.1371/journal.ppat.1010598)
Supplement: Attachment — Submitted filename: Sadasivan et al 2022 Response Letter Revision.docx [file ppat.1010598.s007.docx]

**T H E U N I V E R S I T Y O F B R I T I S H C O L U M B I A**

**Department of Biochemistry and Molecular Biology**

Life Sciences Centre

2350 Health Sciences Mall

Vancouver, B.C. Canada V6T 1Z3

Tel: 604-822-3178

Fax: 604-822-5227

October 20, 2022

Re: PPATHOGENS-D-22-00904

Dear Editors,

We would like to thank you for the decision on our manuscript entitled “Targeting RanBP2/Nup358 by a viral protein inhibits stress granule formation”. We thank the reviewers for their comments and suggestions. In the revised manuscript we have addressed all the comments from the reviewers, including new experimental results and controls that further support our conclusions. Notably, we provide co-IP experiments demonstrating CrPV-1A/NUP358 interactions and qRT-PCR analysis of knockdown experiments. Figures 2, 3, 5, 6, 7, 8, and 9 are modified and contain new experiments/graphs and provide new supplemental Figures S1, S3, S4, S5 and S6We have included the original comments from the reviewers italicized and our response below.

We believe that the manuscript is significantly improved, and we hope that it is acceptable for publication in PLOS Pathogens.

Thank you for your consideration,

Sincerely,

Dr. Eric Jan

Professor

Department of Biochemistry and Molecular Biology

University of British Columbia

Vancouver, Canada

[ej@mail.ubc.ca](mailto:ej@mail.ubc.ca)

**---------------------------------------------------------------------------------------------------------------------**

*Reviewer's Responses to Questions*

***Part I - Summary*** *Please use this section to discuss strengths/weaknesses of study, novelty/significance, general execution and scholarship.*

*Reviewer #1:  The manuscript is well reasoned, well laid out, and the data convincing. The conclusions are well supported by the data; however, the discussion of some of the results could be improved by addressing some of the comments below. Overall, the article is of interest to the PloS Path audience and virology community.*

RESPONSE: We thank the reviewer for the positive feedback.

*Reviewer #2:  The strengths of this work are that it is very interesting, tells an important story, and the data are excellent quality. Comments below are mostly minor, but the authors should address.*

RESPONSE: We thank the reviewer for the positive feedback.

*Reviewer #3: Dr. Jan’s lab has submitted an exciting manuscript that shows a new mechanism by which an RNA virus can modulate the formation of stress granules (SGs). The strengths of the manuscript include 1) a new mechanism showing how viruses can manipulate the formation of stress granules by interfering with proteins associated with the nuclear pore complex and hence nuclear export; 2) showing that RNA viruses that are typically thought to only manipulate processes in the cytoplasm have intimate interactions with the nuclear compartment, and 3) different experimental approaches to dissect the mechanism by which 1A R146A affects SG assembly.

Overall, this manuscript is well written, the experiments are well executed, and the authors have been meticulous in describing the data. The findings described in this manuscript are important to our understanding of how viruses subvert RNA granules in cell to promote virus infection.*

RESPONSE: We thank the reviewer for the positive feedback.

***Part II – Major Issues: Key Experiments Required for Acceptance*** *Please use this section to detail the key new experiments or modifications of existing experiments that should be absolutely required to validate study conclusions.

Generally, there should be no more than 3 such required experiments or major modifications for a "Major Revision" recommendation. If more than 3 experiments are necessary to validate the study conclusions, then you are encouraged to recommend "Reject".*

*Reviewer #1: No major revisions requested, some additional information would be helpful, but not absolutely required (perhaps unless other reviewers also had the same queries), see below.*

RESPONSE: We thank the reviewer for the positive feedback.

*Reviewer #2: No major issues.*

RESPONSE: We thank the reviewer for the positive feedback.

*Reviewer #3: 1. The authors have data showing that 1A protein increases the amount of poly(A)+ RNA in the nucleus. These data are however based on quantification from immunofluorescence images. Since this is one of the data supporting the proposed mechanism, the investigators should include an orthogonal approach to validate these data. This might be undertaken by isolating nuclei and quantifying the amount of poly(A)+ RNA in the GFP-positive cells by RT-qPCR.*

RESPONSE: We thank the reviewer for this insightful suggestion. We are very much interested in determining the identity of the nuclear enriched transcripts in CrPV-1A expressing cells and investigating their role in modulating SG formation as well in virus infection. The ideal experimental design would be to transfect S2 cells with in vitro transcribed CrPV-1A RNAs and then FACS-sort cells, fractionate the nuclear and cytoplasmic compartments, followed by RT-qPCR. However, CrPV-1A expression is limited to a few cells (~5% efficiency) in part due to the low transfection efficiency of the CrPV-1A RNA (16 hrs) and toxicity issues of expressing CrPV-1A, thus the experiment is quite challenging and will require optimization. Our longer time goal will be to further dissect and decouple the toxicity domain of CrPV-1A in order to improve transfection efficiency and cell viability. Moreover, we plan to perform a much more comprehensive study on the nuclear enriched mRNA in CrPV-1A expressing cells by FACS-sorting and fractionating nuclear vs cytoplasm followed by RNA-seq analysis. This global analysis will not only identify the mRNAs that are nuclear enriched but also uncover any effects on pre-mRNA processing. These studies will be included in a future manuscript. At this time, the current model is that the majority of poly(A)+ mRNAs are enriched in CrPV-1A-expressing S2 cells.

*2. Figure 8D and lines 1094-1097: The authors show immunofluourescence data of poly(A)+ RNA and detection stress granules by visualizing Rin protein in foci. There are three concerns with these data.
i) The figure shows some poly(A)+ within nuclei. Here the authors state that in these cells Nup358 is depleted. However, the IFA does not show Nup358 expression/depletion, and a western blot of the depleted protein is not shown. The IFA should include visualization of Nup358 so that cells with increased poly(A)+ in the nucleus can be correctly correlated with Nup358 depletion. Sadasivan and colleagues should also include quantification of the extent of poly(A)+ nuclear localization.*

RESPONSE: We thank the reviewer for pointing this out and we apologize for the confusion of the Nup358 depletion experiments. A western blot showing the depletion of Nup358 was previously included in Figure 9A to show antibody specificity (we showed that Nup358 is knocked-down). We have now moved this figure forward to Figure 8D to avoid confusion.

The following changes have been made to the manuscript: Lines 407-410: We generated an antibody raised against Drosophila Nup358. Immunoblotting for Nup358 showed a distinct protein band that migrated at >245 kDa, which was not detected in Nup358 dsRNA-treated cells, thus showing specificity of the antibody and confirming RNAi-mediated depletion of Nup358 (Fig 8D). Depletion of Nup358 resulted in poly(A)+ signal in the nucleus (Fig 8E,8F), " Updated to show depletion of Nup358 in knockdown

Figure legends for Figure 8D and 9A have been updated.

To our knowledge, the Nup358 antibody that we generated is the only Drosophila antibody available. Unfortunately, the antibody cannot be used for immunofluorescence studies as the antibody also detects non-specific bands (new Figure 8D).

We agree with the reviewer of including the quantification of poly(A)+ in Nup358-depleted cells. The quantification is now shown in the new Figure 8F.

Summary: New Figure 8F, Updated Figure 8D and 9A

*ii) Depletion of Nup358 putatively retains mRNA in the nucleus which then limits the amount of RNA to be redistributed to SGs in the cytoplasm. The authors should show depletion of another similar protein (e.g., GP210) to demonstrate if this effect is a general response or specific to Nup358.*

RESPONSE: We thank the reviewer for this suggestion. We have now added a supplementary figure (Fig S5) showing that depletion of NXF1, a major RNA export factor, also leads to a loss of SGs, supporting the idea that blocking mRNA export is a general response to block SG formation.

The following changes have been made to the manuscript:

Lines 414-417: Added a sentence "Moreover, depletion of NXF1, the main RNA transport factor, resulted in loss of SGs, thus supporting the results that blocking mRNA export, in general, can lead to SG inhibition (Fig S5)”

Summary: New Figure S5

*iii) The authors should include an experiment that rescues the effect by over expressing a RNAi-resistant Nup358.*

RESPONSE: We agree with the reviewer that overexpressing Nup358 in knocked down cells would be ideal. However, it should be noted that the knockdown experiments in Drosophila cells uses 400-600 bp dsRNA. Thus, generating a RNAi-resistant Nup358 would be near impossible. It is possible to generate dsRNAs that target the 3'UTR and then overexpress Nup358, however, this approach will take a considerable amount of time to optimize.

*3. The model figure suggests an interaction between 1A protein and Nup358. While the authors have been cautious to state that this might be a direct or indirect effect, showing such an interaction by co-IP would further support the proposed model by which 1A is affecting SG assembly. Alternatively, Sadasivan and colleagues could perform the same Z-stack microscopy analysis of 1A and lamin in cells depleted of Nup358. The prediction would be that 1A was no longer localized at the nuclear periphery.*

RESPONSE: We thank the reviewer for this suggestion. Unfortunately, the Nup358 antibody that we generated was not ideal for immunoprecipitation experiments, thus we generated a custom FLAG-tagged Nup358 into an expression construct. We first validated the expression of full-length Nup358 by transfecting S2 cells with the construct followed by immunoblotting for Nup358 and FLAG (new Figure S6). We then co-transfected the FLAG-Nup358 construct along with either wild-type or mutant R146A CrPV-1A into S2 cells and performed IP. Wild-type CrPV-1A pulled down FLAG-Nup358 whereas the CrPV-1A (R146A) pulled down Nup358 to a lesser extent (N=3 biological repeats) (new Figure 9A), thus indicating that CrPV-1A interacted with Nup358 in an R146-dependent manner.

The following changes have been made to the manuscript:

Lines 423-437: Add sentences "Our results suggest that the wild type, but not the R146A mutant CrPV-1A manipulates Nup358 to modulate SG formation. We next asked if CrPV-1A interacts with Nup358 and whether this interaction requires the R146 residue. To address this, we generated a *Drosophila* expression construct containing a 3XFLAG tagged-Nup358 (FLAG-Nup358). Transfection of S2 cells for 24 or 48 hours with this construct resulted in immunodetection of Nup358 with either FLAG antibody or Nup358 antibody, thus validating the stable expression of the full-length protein (Fig S6). To address whether CrPV-1A interacts with Nup358, we transfected FLAG-Nup358 in *Drosophila* S2 cells for 24 hours followed by transfection with *in vitro* transcribed wild-type or R146A mutant CrPV-1A-2A-GFP RNA or 5'cap-GFP-poly(A) RNA for 16 hours. Expression of wild-type and mutant R146A CrPV-1A was similar. Immunoprecipitation using anti-FLAG to pulldown FLAG-Nup358 as bait resulted in immunoprecipitation of wild-type CrPV-1A (Fig 9A). By contrast, FLAG-Nup358 immunoprecipitates contained in less mutant CrPV-1A(R146A), indicating that CrPV-1A interacts with Nup358 in an R146-dependent manner (Fig 9A). " to include Immunoprecipitation data

Lines 520-523: Updated the sentence " Moreover, all of these effects are dependent on the R146 residue within the C-terminal tail of CrPV-1A. The R146A mutation may disrupt CrPV-1A/Nup358 interactions (Fig 9) or may alter CrPV-1A subcellular localization and thereby sequester it from interacting with Nup358" in the discussion

Summary: New Figure S6 and Figure 9A, Updated Materials and methods (line 592-595)

*4. Figure 7: from the figure legend it seems that the data should show images from mock- CrPV-WT and CrPV-R146A virus infections. The image panel does not represent this description. The figure should also include quantification of the amount of poly(A)+ in the nucleus.*

RESPONSE: We apologize for the confusion. Figure 7A only shows NXF1-depleted cells (no infection). As suggested by the reviewer, quantitation of poly(A)+ RNA accumulation is now included in the new Figure 7B, demonstrating that NXF1 KD leads to nuclear enrichment of poly(A)+ mRNA.

The following changes have been made to the manuscript.

Line 372: Updated the sentence "Incubation of S2 cells with NXF1 dsRNA but not control GFP dsRNA resulted in accumulation of nuclear poly(A)+ mRNA indicating that NXF1 knockdown was efficient in impairing mRNA export (Fig 7A-B)" to cite Figure 7B. The figure legend has been modified accordingly.

Summary: New Figure 7B

*5. Line 385: “Knockdown of Rae1, mTor, Nup88 or Nup214 did not restore VP2 expression under mutant CrPV (R146A) virus infection compared to wild-type infection” The authors do not specifically state the number of times that the quantitation of the western blot of VP2 was performed but looking at the error bars in Figure 8B, it seems that mTor does restore infection of the CrPV (R146A) virus. Undertaking RT-qPCR to determine the relative amount of viral RNA in these knockdown cells would clarify this.*

RESPONSE: We thank the reviewer for pointing out this. We have updated the figure legend to include the number of replicates. The data shown are from at least three independent experiments.

We agree with the reviewer that quantification of the KD experiments would help with interpretation. The extent of KD by qRT-PCR is now shown in Figure S3. Of note, the knockdown of some mRNAs was moderate. Specifically, qRT-PCR of Nup358 KD shows ~50% knockdown. yet Western blotting for Nup358 shows nearly complete KD (new Figure 8D). We will certainly follow up on some of these candidates in our future studies, possibly likely using Western blotting and/or proteomic analysis to ensure that KD is complete at the protein level.

*6. Figure 8: The authors use RNAi to deplete a subset of proteins involved in mRNA export, yet no experiments are provided to validate the depletion of the proteins or at least depletion of transcript levels.*

As discussed above, we validated the knockdown experiments by qRT-PCR shown in Figure S3. Currently, no antibodies are available for all of these proteins (to our knowledge). Thus, we generated our own antibodies to Nup358. Further validation at the protein level will require generating antibodies and/or using proteomics.

The following changes have been made to the manuscript.

Line 387-390: Updated the sentence "qRT-PCR analysis confirmed that specific mRNAs were depleted by dsRNA treatment as compared to the control (dsFLuc) treated cells (Fig S3)”

Summary: New Figure S3

*7. The formation of SGs formation is assayed following PatA treatment in cells that by western blot show efficient depletion of Nup358. However, CrPV infection only causes a 50% decrease in Nup358. It would be good to correlate the extent of Nup358 turnover with the extent of SG formation/inhibition?*

RESPONSE: We thank the reviewer for this insightful comment. We agree that it would be informative to deplete Nup358 to certain levels (i.e. 25%, 50%, 75% depletion) to determine the effects on SG formation and nuclear enrichment of poly(A)+ mRNAs. However, this approach would be quite challenging. In CrPV-infected cells, it is probable that several viral proteins are modulating several cellular processes to promote infection. Our studies point to the depletion of Nup358 as a contributing factor to one of these processes and may in fact be a major factor given that a single R146 mutation can abolish several effects by CrPV-1A (i.e. mRNA enrichment, Nup358 depletion, mRNA transcriptome, etc).

*8. Why does MG132 decrease VP2 levels? This is a curious observation, and it would be useful if the authors discussed this further in the discussion section.*

RESPONSE: We thank the reviewer for pointing this out. CrPV inhibits RNAi by targeting Ago2 for proteasome-mediated degradation. Therefore, inhibition of proteasome is expected to have a negative impact on virus infection via several cellular processes. Similar effects were shown in other virus infections: MG132 treatment decreased poliovirus and Vesicular stomatitis virus infection (PMID: 18382670). The virus depends on the proteasome pathway for facilitating infection.

The following changes have been made to the manuscript:

Lines 516-519: Added a sentence " We noted that MG132 treatment decreases CrPV infection in general, which is similar to that observed with other plus-strand RNA virus infections (85). It is likely that proteasome activity is required for CrPV infection. For example, Ago2 degradation by CrPV-1A requires proteasome activity (59). " on the effect of MG132 on VP2 levels in the discussion.

*9. In Figure 3 Sadavisan et al., show that R146A affects the proteolytic processing of the polyprotein. It is disappointing that the authors only briefly mention this observation or the effect of this defect in the discussion. It would seem that decreased 2A proteolytic processing could have important effects on SG formation, nuclear accumulation of poly(A)+ RNA or degradation of Nup358.*

RESPONSE: We thank the reviewer for pointing this out and we agree! The effects of the precursor protein are currently being investigated and we anticipate that a more comprehensive study (including the effects on 2A activity per se) will be included in a future manuscript. On page 26, lines 543-544, we now include discussion of the possibility of the minor fusion protein that may have an impact on mRNA export, Nup358 degradation and SG formation.

***Part III – Minor Issues: editorial and Data Presentation Modifications*** *Please use this section for editorial suggestions as well as relatively minor modifications of existing data that would enhance clarity.*

*Reviewer #1: (No Response)*

*Reviewer #2: 1. Fig. 3A. page 13, line 257: the text states the individual CrPV-1A proteins were detected at similar levels after transfection. However, their levels seem to vary quite a bit, e.g., L17A is significantly lower than F114A, R146A, and WT. Could the authors clarify their statement?*

RESPONSE: We have clarified this statement:

Lines 257-261: Updated with the sentence " For most mutant CrPV-1A, the individual CrPV-1A and GFP proteins were detected at similar levels after transfection, indicating that the wild-type and mutant CrPV-1A proteins are expressed and processed efficiently by the CrPV-2A ‘stop-go’ translation activity (>99% efficiency). However, mutant L17A and A21D CrPV-1A were expressed at lower levels, suggesting that these mutations affected the stability of the proteins "

*2. Fig. 3A, page 13, line 261: the authors note that a longer exposure reveals an unprocessed 1A-2A-GFP protein for the 1A (R146A) mutant. However, this must be a different blot since there is a gap between GFP lanes 7 and 8 in the lower exposure that is missing from the longer exposure. Please clarify.*

RESPONSE: Thank you for bringing this to our attention. We apologize for including figures from two different replicates. Figure 3A has been updated to show the same blot.

*3. Fig. 7A: the labels are reversed for the Hoechst and Poly A images. Additionally, the Hoechst image for the GFP dsRNA control is very faint compared to the NXF1 dsRNA image.*

RESPONSE: We apologize for the confusion. The poly(A) was shown in blue and Hoechst in grey in the previous version. We have updated the image (Figure 7A) and colored Hoechst in Blue and Poly(A) in Cyan to keep the colors consistent with other figures. The figure legend has been modified accordingly.

*4. Fig. 7B: authors should show efficiency of protein reduction by Western blot following dsRNA-mediated knockdowns.*

RESPONSE: As there are no antibodies available for the Drosophila proteins, we confirmed knockdown by monitoring poly(A)+ RNA accumulation in the nucleus of NXF1 knockdown cells. The quantitation of poly(A)+ RNA accumulation is now included in the new Figure 7B.

The following changes have been made to the manuscript.

Line 372: Updated the sentence " Incubation of S2 cells with NXF1 dsRNA but not control GFP dsRNA resulted in accumulation of nuclear poly(A)+ mRNA indicating that NXF1 knockdown was efficient in impairing mRNA export (Fig 7A,7B)" to cite Figure 7B

*5. Fig. 8A: the authors should show efficiency of protein reduction by Western blot following dsRNA-mediated knockdowns.*

RESPONSE: As we responded above, there are no antibodies available for the Drosophila proteins, so we performed qRT-PCR to validate the knockdown, which showed reduction of the target mRNAs compared to the control dsFLuc treated cells in the new Figure S3.

The following changes have been made to the manuscript.

Line 387-390: Updated the sentence "qRT-PCR analysis confirmed that specific mRNAs were depleted by dsRNA treatment as compared to the control (dsFLuc) treated cells (Fig S3)”

*6. Fig. 8C, page 19, line 395: if there is decreased viral RNA with GP210 knockdown for R146A (panel C), could the authors explain the comparable levels of VP2 protein upon GP210 knockdown with WT and R146A (panel B).*

RESPONSE: We thank the reviewer for pointing this out. In this screen, we are monitoring one-step growth and it is possible that the GP210 might play another role during virus infection (eg: translation vs. replication). It would be interesting to monitor viral RNA and protein levels in a longer time course. This is something that we are following up on in our future studies.

*Reviewer #3: 1. Line 121: Misalignment of formatting of references “termed antiviral SGs (avSGs)6776,”*
*2. Line 223: period missing at the end of the sentence*
*4. Line 1097: A period is missing at the end of the sentence. “The arrows show Nup358 knockdown cells.”*

RESPONSE: We thank the reviewer for pointing these out and they have been fixed.

*3. Figure 2: It would be good to include data from the WT in Figure 2 so that the rescue effect of mutations within the BC box can be more easily compared*

RESPONSE: We thank the reviewer for this. We have updated the graph to include the wild-type controls.
